# Divide and conquer? Size adjustment with allometry and intermediate outcomes

**DOI:** 10.1186/s12915-017-0448-5

**Published:** 2017-11-09

**Authors:** Shinichi Nakagawa, Fonti Kar, Rose E. O’Dea, Joel L. Pick, Malgorzata Lagisz

**Affiliations:** 1 Evolution & Ecology Research Centre and School of Biological, Earth and Environmental Sciences, University of New South Wales, Sydney, NSW 2052 Australia; 2 Diabetes and Metabolism Division, Garvan Institute of Medical Research, 384 Victoria Street, Darlinghurst, Sydney, NSW 2010 Australia

## Abstract

Many trait measurements are size-dependent, and while we often divide these traits by size before fitting statistical models to control for the effect of size, this approach does not account for allometry and the intermediate outcome problem. We describe these problems and outline potential solutions.

**Electronic supplementary material:**

The online version of this article (doi:10.1186/s12915-017-0448-5) contains supplementary material, which is available to authorized users.

## Adjusting for size in statistical analysis

Biologists measure traits of organisms, characterizing a range of features including morphology, physiology and behaviour. Many of these traits are size-dependent. For example, larger animals eat more and larger plants absorb more than smaller counterparts. However, when size is not our trait of interest, we often want to know values of focal traits after controlling for the effect of size. An intuitive way to account for organismal size is to divide a trait of interest by size (for example, the amount of food consumed or nutrient absorbed divided by mass or length). This method (hereafter called the “division” method) generates size-adjusted trait values, which can be used for statistical analyses. Indeed, the use of the division method is prevalent in the literature [1–4], but is it correct? The division method poses two major problems because, first, we assume a linear relationship between adjusted traits and size and, second, an experimental treatment often affects not only a trait of interest but also other variables, such as size. Here, we deliberate on these two problems and provide potential solutions.

## Allometric scaling of organismal traits

The power law (meaning non-linear) relationship between body size and body parts is believed to have been first described by the evolutionary biologist Julian Huxley, (a grandson of “Darwin’s bulldog” Thomas Huxley) on the basis of his study of claw size and body size in fiddler crabs [5]. This non-linear relationship was later termed “allometry” (meaning “different measure”) [6]. Huxley’s original study was on ontogenetic allometry—the relationship between two traits while an organism is growing [7]. This type of allometry is distinguished from the two other types: evolutionary allometry and static allometry [7]. Evolutionary allometry concerns between-species variation in the relationship between traits (see [8] for an in-depth review), whereas static allometry concerns the relationship between two traits of mature individuals from the same species. Here, we focus mainly on static allometry.

**Fig. 1.**
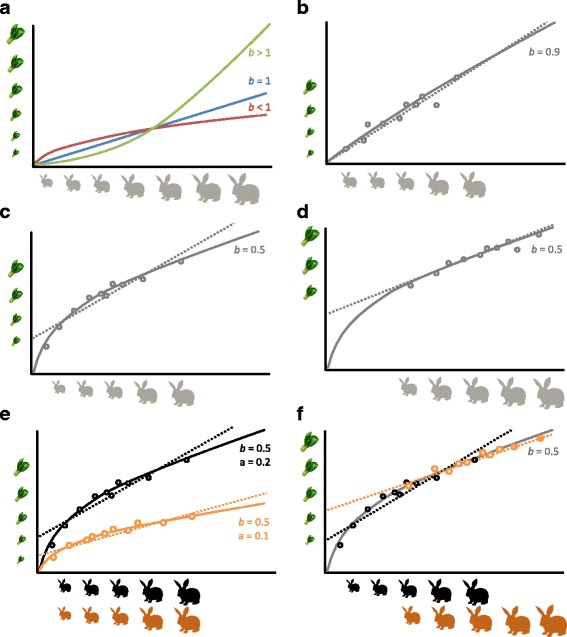
Conceptual plots for allometric relationships. **a** Three different types of allometric relationships between food intake (a focal trait; on the *y* axis) and size (on the *x* axis) with different exponents, *b*, and a fixed slope, *a* (Eq. 1); note that when *b* = 1, the relationship is linear. **b** When *b* is close to 1 (*b* = 0.9), the relationship becomes nearly linear without log-transformation (*dotted line*). **c**, **d** Even when *b* is not close to 1 (*b* = 0.5), whether the relationship is non-linear depends on how the data are distributed; the non-linear relationship in **d** could be much better approximated by a linear line than that in **c. e**, **f** Notably, the same slopes (*b* = 0.5) can be estimated as having different slopes if not log-transformed due to having different values for *a*, as in **e**, or being on different parts of a non-linear curve, as in **f**

Historically, allometric studies have focused on morphological traits. However, physiological traits (such as cellular and drug metabolism [9, 10]) and behavioural traits (for instance, food consumption [2, 11]) also scale allometrically with organismal size. Traits follow the power law relationship described by Huxley [5], which is expressed as:

1 $$ {y}_{\left[\mathrm{trait}\right]}=a{x}_{{\left[\mathrm{size}\right]}_b}, $$

where *y*_[trait]_ is the focal variable (trait), *x*_[size]_ is the size variable, and *a* and *b* are constants (parameters) estimated from data (Fig. 1a). In order to linearize this non-linear equation, we can take the natural logarithm of both sides; thus, we get:

2 $$ {y}_{\left[\ln \left(\mathrm{trait}\right)\right]}=\ln a+b{x}_{\left[\ln \left(\mathrm{size}\right)\right]}, $$

where *y*_[ln(trait)]_ is ln(*y*_[trait]_) and *x*_[ln(size)]_ is ln(*x*_[size]_), and ln is the log_*e*_ transformation (see [12, 13] for discussions of allometry and the log transformation).

Now consider an experimental study that compares a size-dependent trait of two groups (for example, control vs. treatment; note that non-experimental groups—such as males vs. females, or two natural populations—could be also used). When we use the division method to adjust for size in the analysis, we use the following linear model:

3 $$ {y}_{\left[\mathrm{trait}/\mathrm{size}\right]i}={b}_0+{b}_1{x}_{\left[\mathrm{group}\right]i}+{e}_i, $$

where *y*_[trait/size]_ is a variable derived from the focal trait divided by size, *x*_[group]_ is a “dummy variable”, which takes the value 0 or 1 to indicate the presence or absence of a particular effect in order to sort data in mutually exclusive groups (for instance, 0 = control and 1 = experiment), *b*_0_ is the intercept, *b*_1_ is the slope (or in this case, the difference between the control and experimental group: experimental or treatment effect), *e* is the residual (error) term (which represents deviations from the regression line), and the subscript *i* indicates the *i* ^*th*^ value (*i* = 1…*n*, *n* = sample size; this linear model, Eq. 3, is equivalent to a *t*-test comparing the two groups). However, this model is not ideal because the division method (Eq. 3) creates a ratio variable (*y*_[trait/size]_), the distributional properties of which may not meet an important assumption of a linear model: the residuals are normally distributed [1]. Furthermore, we are not able to estimate the allometric scaling exponent *b*.

We can improve Eq. 3 by fitting size as a predictor variable because it avoids creating a ratio variable in the response variable. Then, we have:

4 $$ {y}_{\left[\mathrm{trait}\right]i}={b}_0+{b}_1{x}_{\left[\mathrm{group}\right]i}+{b}_2{x}_{\left[\mathrm{size}\right]i}+{e}_i, $$

where *b*_2_ is the slope for size, and the other symbols are as above. This approach is again common in the biological literature. However, both Eq. 3 (the division method) and Eq. 4 assume a linear relationship between a trait and size; they ignore the allometric relationship shown in Eqs. 1 and 2. Therefore, a better statistical model would be to linearize the equation by taking natural logs:

5 $$ {y}_{\left[\ln \left(\mathrm{trait}\right)\right]i}={b}_0+{b}_1{x}_{\left[\mathrm{group}\right]i}+{b}_2{x}_{\left[\ln \left(\mathrm{size}\right)\right]i}+{e}_i, $$

where *b*_0_ and *b*_2_ correspond to ln*a* and *b* in Eq. 2, respectively, and the other symbols are as above. It is also notable that Eqs. 4 and 5 could produce comparable results, depending on the exponent of the power law relationship, *b*, and the distribution of trait data (Fig. 1b–d). However, Eq. 4, like Eq. 3, could lead not only to a spurious treatment effect, *b*_1_ [2], but also to spurious interactions (which means that the control and experimental groups have different slopes, *b*_2_ as described in Fig. 1e, f); see the next section for modeling the interaction (that is, different slopes). However, it turns out that even Eq. 5 can provide an incorrect estimate of *b*_1_.

## Experimental treatments and intermediate outcomes

**Fig. 2.**
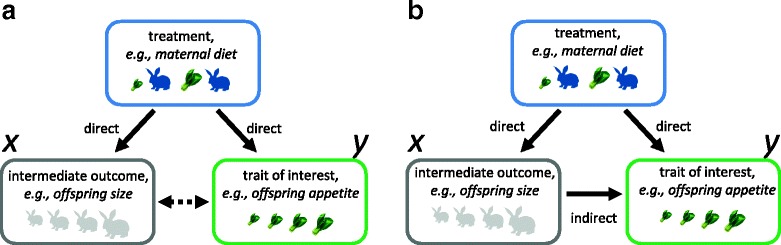
Two scenarios of the relationship among an experimental treatment, a trait of interest (focal variable, *y*) and an intermediate outcome (*x*). **a** The treatment affects both *x* and *y*, and therefore *x* and *y* are correlated (*dotted line* with a *double-headed arrow*) but *x* does not affect *y*. **b** The treatment affects both *x* and *y*, and then *x* also affects *y*

An experimental treatment is intended to change a focal variable, but it often affects other unintended variables, referred to as mediators or intermediate outcomes [14]. For example, maternal dietary conditions, such as dietary restriction or over-nutrition, may influence offspring size (*x*), as well as offspring food intake (the focal trait; *y*) [2, 11]. If we know that offspring body mass and offspring food intake are correlated, we may want to account for the effect of offspring size when assessing experimental effects on offspring food intake. However, as both offspring body mass and food intake are measured after the treatment has been applied (offspring body mass cannot be measured before maternal diet is manipulated), we do not know the chain of causation. Imagine two scenarios. In scenario A, offspring body mass and food intake are mechanistically linked, and the maternal diet treatment subsequently affects both traits (direct effects; Fig. 2a). Alternatively, in scenario B, the treatment affects offspring body size (direct effect), which then influences offspring food intake (indirect effect), as well as a potential direct effect of the treatment on offspring food intake (Fig. 2b).

In both scenarios, we would observe a treatment effect in both *x* and *y*, but when we correct for *x* (as in Eq. 5), the direct effect of the treatment on *y* could diminish, disappear or even reverse (known as Lord’s paradox) [15]. If scenario A is true, then correcting for *x* leads to an underestimation of the direct treatment effect (*b*_1_ in Eq. 5) on *y* (known as over-adjustment bias) [14, 16]. If scenario B is true, then not correcting for *x* leads to an overestimation of the direct effect on *y* (specifically, direct effects plus indirect effects). Unfortunately, we are unlikely to disentangle the two scenarios unless we have prior knowledge of the mechanistic underpinnings of these relationships. Furthermore, even if we know that scenario B is true, we must assume that all subjects’ size responds in the same way to the treatment (for example, every subject gains 200 g) to obtain the correct *b*_1_ (the direct effect) using Eq. 5, although this seems unlikely. Therefore, it has been suggested that we should not add an intermediate outcome (such as size) to the model regardless of the scenarios [14], as follows:

6 $$ {y}_{\left[\ln \left(\mathrm{trait}\right)\right]i}={b}_0+{b}_1{x}_{\left[\mathrm{group}\right]i}+{e}_i, $$

where the symbols are as above. Then, at least we will get the total (direct and indirect) effect on *y* as the experimental effect, *b*_1_ (for further discussion of intermediate outcomes and their problems see [14, 16–18]).

**Fig. 3.**
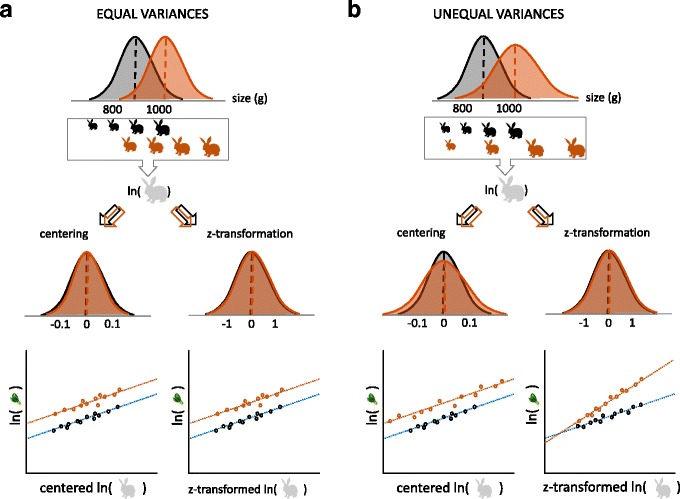
Visualizations of within-group centering and *z*-transformation. **a** Within-group centering of a size variable with two groups (*black*, control; *orange*, experimental) with the same variances, and **b** within-group *z*-transformation of a size variable with two groups with different variances

Importantly for our discussion here, this solution means that we are unable to estimate the allometric scaling component. Here we suggest a workaround using a linear model [19] (see reference [20] for an example), which can be written as:

7 $$ {y}_{\left[\ln \left(\mathrm{trait}\right)\right]i}={b}_0+{b}_1{x}_{\left[\mathrm{group}\right]i}+{b}_2{x}_{\left[\mathrm{wgc}\left(\ln \left(\mathrm{size}\right)\right)\right]i}+{e}_i, $$

where the subscript “wgc” stands for within-group centering, which adjusts values (ln(size)) for two (or more) different groups separately by setting respective group means as zero (Fig. 3). Figure 3a visualizes what such centering does to a size variable; the order of the two transformations for *x*_[wgc(ln(size))]_ (log first, then center) is particularly important, as one cannot take the logarithm of negative values. The within-group centering separates the experimental effect on size (indirect effect), so that the indirect effect is now absorbed into *b*_1_. With this approach, we can model an allometric relationship, although we cannot obtain a “size-corrected” experimental effect (an unbiased direct effect, but see [21, 22] for a potential issue and solution; Fig. 2). Note that the estimates of *b*_1_ from Eqs. 6 and 7 are the same, as are the estimates of *b*_2_ (that is, the allometric scaling exponent) from Eqs. 5 and 7.

Another way to do such adjustment is through *z*-transformation instead of centering, which scales distributions of ln(size) for both the control and experimental group to have the mean of 0 and standard deviation of 1. These two methods (centering and z-transformation) are equivalent when the variances of the two groups are the same.

However, if slopes differ between the two groups after *z*-transformation, it should be checked whether the transformation caused the significant differences, which may happen if the variances for ln(size) to differ between the two groups. (Figure 3b; see below for how to detect differences in slopes). The choice of transformation to use (*z*-transformation or centering) should not affect the experimental effect, but *z*-transformation could lead to confusion if one is interested in obtaining allometric parameters (*a* and *b* in Eqs. 1 and 2). Centering size on the log scale may also be easier to interpret than *z*-transformation (the issue of different variances described above aside). However, *z*-transformation has some advantages over centering when applied normally to a predictor variable (meaning, not within-group transformations). For example, *z*-transformation can help another aspect of interpretation because regression coefficients of continuous variables become comparable (in that they are standardized beta coefficients) [23].

Notably, we still assume that the variances for the response variable are homogenous in this model (in fact, this is true for all the models above). When this assumption is not met (that is, the response variable, *y*, is heteroscedastic), either we can model different variances in the response between two groups, or we can use “robust” statistical estimators, which take heteroscedasticity into account (for details on how, see [24]). We suspect that when variance in body sizes between two groups is different, it is likely that a trait of interest is heteroscedastic.

Equations 5 and 7 only model a change in the intercepts between two groups, which corresponds to a change in ln(*a*) in Eq. 2 (Fig. 3a). It is possible that some experimental treatments could affect the slope, which corresponds to a change in the exponent *b* in Eqs. 1 and 2. Biologically, this parameter, *b*, is less likely to change than *a*, at least for some allometric relationships (for an example see reference [10]). Nonetheless, we should probably check for such a change. An equation that models different slopes between two groups can be written as:

8 $$ {y}_{\left[\ln \left(\mathrm{trait}\right)\right]i}={b}_0+{b}_1{x}_{\left[\mathrm{group}\right]i}+{b}_2{x}_{\left[\mathrm{wgc}\left(\ln \left(\mathrm{size}\right)\right)\right]i}+{b}_3{x}_{\left[\mathrm{group}\right]i}{x}_{\left[\mathrm{wgc}\left(\ln \left(\mathrm{size}\right)\right)\right]i}+{e}_i, $$

where *b*_3_ is the difference in slopes between the control and experimental groups, and *x*_[group]_ *x*_[wgc(ln(size))]_ is an interaction term between *x*_[group]_ and *x*_[wgc(ln(size))]_ (Figs. 1 and 3). An example of implementing the above procedures in the statistical environment *R* [25] is provided in the Additional file 1. In this supplement, we refer to the assumption that size is measured without error in linear models such as Eqs. 4, 5, 7 and 8 [26, 27] and also provide a solution to this problem when this assumption is not met.

## Divide and conquer? No, leave them alone!

We have described the two major shortcomings of the division method, so far focusing on scenarios when we use the ratio variable (for instance, dividing a focal trait by size) as a response variable (*y*). However, it is just as common to find the ratio variable being used as a predictor variable (*x*). Among the many issues of using ratios as predictors, there is one problem that is very general and straightforward to describe [1]. When we have two variables (or traits), A and B, their ratio is A/B. The variable A/B fitted as a predictor can be considered an interaction term, because A/B can be re-expressed as AB^−1^ (compare it with *x*_[group]_ *x*_[wgc(ln(size))]_ in Eq. 8). Therefore, we should also fit A and B^−1^ as predictors (main effects), along with the interaction term (AB^−1^) [1]. More generally, it is usually not advisable to create and fit a derived variable (in other words, a variable comprised of more than one variable, such as, A/B, AB^2^) to a model. For linear modelling, raw measurements or their direct transformations should be used to control for confounding effects. Finally, because the division method and other inappropriate modelling procedures could lead to spuriously significant results and/or biased effect size estimates [1, 2], correct modelling practice is essential to avoid exacerbating the current “reproducibility crisis” [28, 29].

## Additional file

Additional file 1: Supplementary material. (PDF 320 kb)
