# Supplementary material for: Divide and conquer? Size adjustment with allometry and intermediate outcomes
Source: BMC Biol. 2017 Nov 9;15:107. doi: 10.1186/s12915-017-0448-5 (PMC5679152; doi:10.1186/s12915-017-0448-5)
Supplement: Supplementary file 1 — Supplementary material. (PDF 320 kb) [file 12915_2017_448_MOESM1_ESM.pdf]

Divide and conquer? Size adjustment with allometry and intermediate outcomes

Supplementary Material

Shinichi Nakagawa, Fonti Kar, Rose E. O'Dea,
Joel L. Pick and Malgorzata Lagisz

October 17, 2017

To demonstrate the problems described in the paper, using *R* we will analyze some simulated data of an experimental treatment on maternal diet, and its effect on offspring food intake, as well as a possible mediating factor - offspring body mass.

1 Division Method and Allometry

First, we will look at offspring food intake. We can see that there is a clear relationship between this and offspring mass.

```
library(scales)
plot(food_intake ~ mass, pch = 19, cex = 0.5, col = alpha(1, 0.5))
```

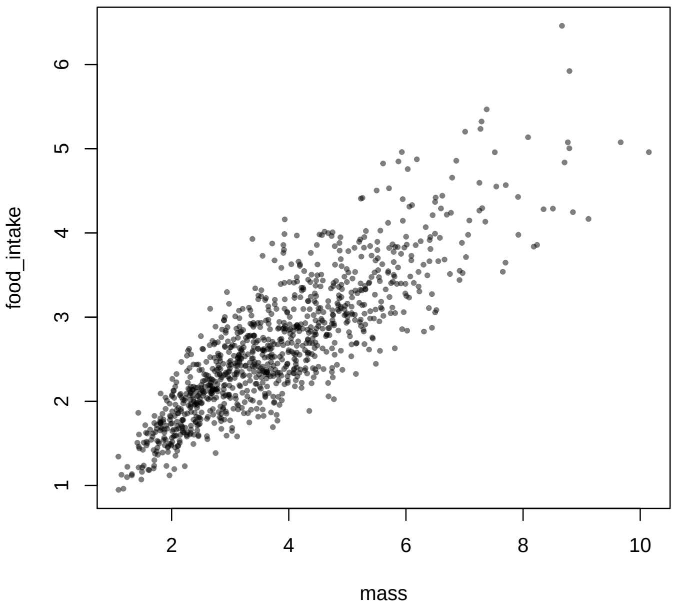

When estimating the experimental effect of maternal diet on offspring food intake, we may therefore want to correct for offspring body mass. This is usually done through the "division method", as in Equation 3:

```
mod_E3 <- lm(food_intake/mass~treatment)
summary(mod_E3)$coef

##                Estimate  Std. Error   t value      Pr(>|t|)
## (Intercept)  0.8310806 0.005092556 163.19519  0.000000e+00
## treatment   -0.1927299 0.007201961 -26.76075 2.336256e-119
```

Here we find a strong negative effect of the treatment on offspring food intake. However, this makes certain strong assumptions about the relationship between food intake and mass. We can relax these slightly by including mass as a covariate, as in Equation 4:

```
mod_E4 <- lm(food_intake~mass+treatment)
summary(mod_E4)$coef

##                Estimate Std. Error   t value      Pr(>|t|)
## (Intercept)  0.7815235 0.03529852  22.14040  1.165119e-88
```

```
## mass          0.5416657 0.01070317  50.60798 1.088619e-277
## treatment   -0.3777156 0.03193439 -11.82786  2.671093e-30
```

Note that, although in both models the treatment effect is significant, the estimate changes substantially. When visualising this data, it appears as though the relationship between offspring food intake and body mass is different between the two treatments:

```
plot(food_intake ~ mass, pch = 19, cex = 0.5, col = alpha(c(1, 2)[as.factor(treatment)],
    0.5))
```

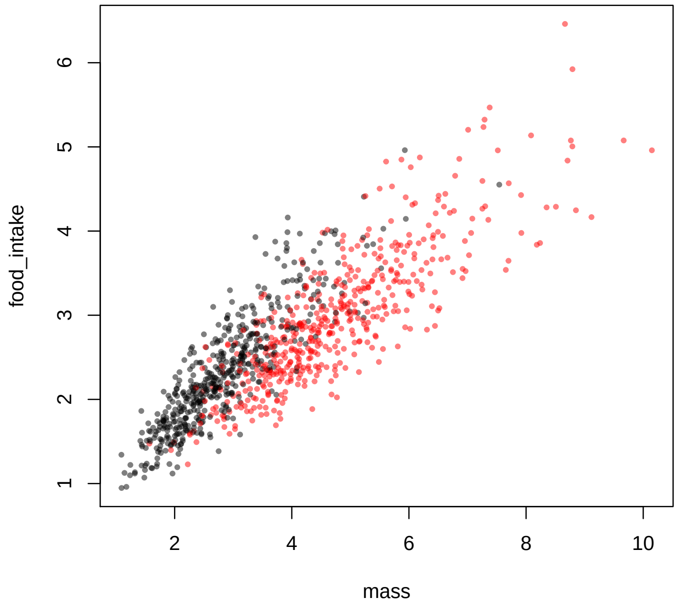

Testing this, we indeed find support for it:

```
mod_E4_interaction <- lm(food_intake~mass*treatment)
summary(mod_E4_interaction)$coef

##                   Estimate Std. Error   t value      Pr(>|t|)
## (Intercept)      0.4790247 0.05609203  8.539979  4.967450e-17
## mass             0.6475470 0.01868291 34.659855 2.541929e-173
## treatment        0.1533394 0.08366893  1.832692  6.714679e-02
## mass:treatment -0.1542969 0.02255347 -6.841380  1.367601e-11
```

These analyzes, however, ignore the possible allometric relationship between the two variables, which we can account for by log transforming both x and y variables, as in Equation 5:

```
plot(food_intake ~ mass, log = "xy", pch = 19, col = alpha(c(1, 2)[as.factor(treatment)],
    0.5), cex = 0.5)
```

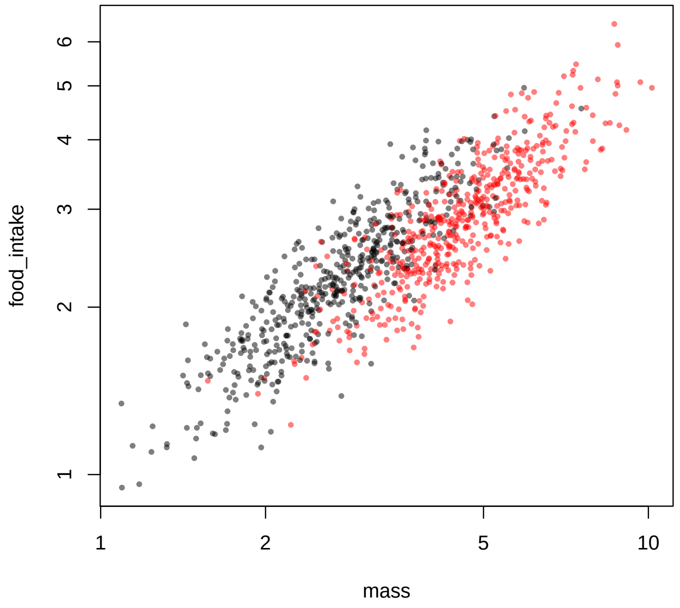


```
mod_E5 <- lm(log(food_intake) ~ log(mass) + treatment)
summary(mod_E5)$coef

##                 Estimate Std. Error     t value      Pr(>|t|)
## (Intercept)  0.004857032 0.01592124   0.3050661  7.603794e-01
## log(mass)    0.798599971 0.01464055  54.5471440 1.538920e-301
## treatment   -0.161459536 0.01162303 -13.8913495  3.091704e-40
```

When analyzed this way, we find that there is in fact no difference in the slope between the two methods, as in Equation 8:

```
mod_E8 <- lm(log(food_intake)~log(mass)*treatment)
summary(mod_E8)$coef

##                         Estimate Std. Error    t value      Pr(>|t|)
```

```
## (Intercept)          -0.00818848 0.02065916 -0.3963607  6.919239e-01
## log(mass)             0.81165522 0.01969566 41.2098611 1.894665e-217
## treatment            -0.12404981 0.03950042 -3.1404677  1.736499e-03
## log(mass):treatment -0.02917783 0.02944451 -0.9909431  3.219540e-01
```

This interaction was, in fact, an artifact of not accounting for the underlying allometric relationship (see Figure 1F).

2 Intermediate Outcomes

We are assuming here that the experimental treatment on maternal diet acted on offspring food intake through its effect on offspring mass (indirect effect), as well as the direct effect (Figure 2B). However, the treatment was applied before both variables were measured, meaning the causal links between the variables is not known. In other words, the treatment effect may also have occured on top of the relationship between the two offspring traits, i.e. there is no indirect effect of the treatment on offspring food intake effect mediated by offspring mass (Figure 2A). Indeed, if we just test for the effect of maternal treatment on offspring food intake, we get a very different picture of the effect, as in Equation 6:

```
boxplot(log(food_intake)~treatment, ylab="Food intake")
```

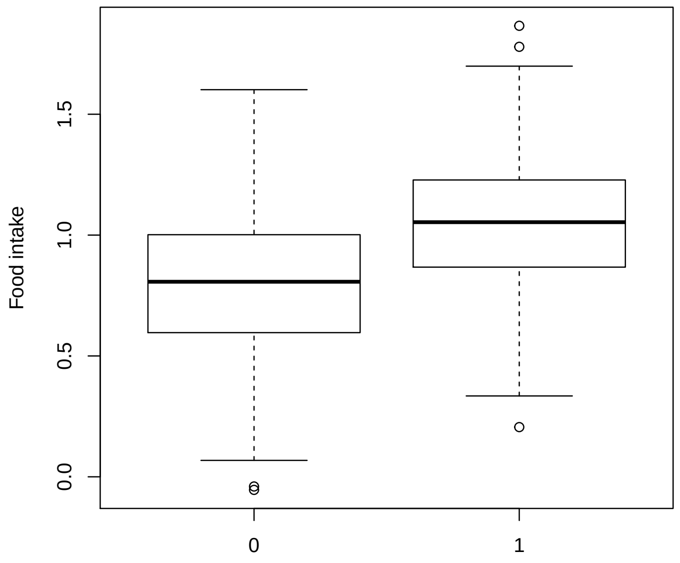


```
mod_E6 <- lm(log(food_intake)~treatment)
summary(mod_E6)$coef

##              Estimate Std. Error  t value     Pr(>|t|)
## (Intercept) 0.8028611 0.01253257 64.06198 0.00000e+00
## treatment   0.2473693 0.01772373 13.95696 1.42252e-40
```

Now we find a strong positive effect of the treatment on offspring food intake. This reversal of effect is known as Lord's paradox. In this model, however, we do not get an estimate of the allometric scaling exponent. We can get this through using within-group centering, as in Equation 7:

```
## function to do within group centering and scaling
groupScale <- function(formula, data=NULL, center=TRUE, scale=FALSE){
  if(is.null(data)) data <- model.frame(formula)
  scaled <- rep(NA,nrow(data))    #empty vector
  for(i in  unique(data[,2])){
    elements <- which(data[,2]==i)
    scaled[elements] <- scale(data[elements,1], scale=scale, center=center)
  }
  return(scaled)
```

```
}

logMass_scaled <- groupScale(log(mass)~treatment)
mod_E7 <- lm(log(food_intake)~logMass_scaled+treatment)
summary(mod_E7)$coef

##                 Estimate  Std. Error   t value      Pr(>|t|)
## (Intercept)    0.8028611 0.006281731 127.80890  0.00000e+00
## logMass_scaled 0.7986000 0.014640546  54.54714 1.53892e-301
## treatment      0.2473693 0.008883709  27.84527 1.06644e-126
```

Note that the estimate for the treatment effect here is the same as the last model,

```
coef(mod_E7)["treatment"];coef(mod_E6)["treatment"]

## treatment
## 0.2473693
## treatment
## 0.2473693
```

and the estimate for the effect of mass (i.e. the allometric scaling exponent) is the same as above.

```
coef(mod_E7)["logMass_scaled"]; coef(mod_E5)["log(mass)"]

## logMass_scaled
##         0.7986
## log(mass)
##    0.7986
```

3 Measurement Error

Researchers may want to account for measurement error in the predictor variable (i.e. body mass) as well as in the response. The assumption in the models described above

is that body mass (x) is measured without error. The presence of measurement error in predictors acts to downwardly bias the estimated slope.

This can be accounted for by using a bivariate model. This estimates the residual variance (σ^2) and covariance ($\sigma_{y,x}$) of body mass (x) and food intake (y) (i.e. assumes that there is error in both), as the variance-covariance matrix

$$\begin{bmatrix} \sigma_y^2 & \sigma_{y,x} \\ \sigma_{y,x} & \sigma_x^2 \end{bmatrix} \tag{1}$$

From this, we can derive the slope of the regression of y on x

$$\frac{\sigma_{y,x}}{\sigma_x^2} \tag{2}$$

In the context of what has already been discussed, the bivariate model assumes the second scenario, i.e. that body mass and food intake are correlated, and that the treatment may subsequently affect both traits (Figure 2A). The model is therefore analagous to Equation 7. We can run a bivariate model in the *MCMCglmm* package in *R*

```
library(MCMCglmm)
dat <- data.frame(logFood = log(food_intake), logMass = log(mass), treatment)
mod_E7_MV <- MCMCglmm(cbind(logFood, logMass) ~ trait + treatment:trait - 1,
    rcov = ~us(trait):units, data = dat, family = c("gaussian", "gaussian"),
    verbose = FALSE)
```

First we can look at the estimated variance covariance matrix

```
matrix(summary(mod_E7_MV)$Rcovariances[, "post.mean"], nrow = 2, dimnames = list(c("y",
    "x"), c("y", "x")))

##            y          x
## y 0.07866071 0.07375522
## x 0.07375522 0.09236607
```

and then estimate the slope. The advantage of using Baysian methods for a calculation like this is that we can do it over the estimates from all of the iterations, and so have an error distribution for the derived estimate.

```
mod_E7_MV_slope <- mod_E7_MV$VCV[, "traitlogFood:traitlogMass.units"]/mod_E7_MV$VCV[,
    "traitlogMass:traitlogMass.units"]
```

We can see that this almost identical to that calculated above from Equation 7

```
c(mean = mean(mod_E7_MV_slope), SE = sd(mod_E7_MV_slope))

##       mean         SE
## 0.79853118 0.01475429

summary(mod_E7)$coef["logMass_scaled", 1:2]

##   Estimate Std. Error
## 0.79859997 0.01464055
```

In this model we jointly estimate the treatment effect on x and on y:

```
summary(mod_E7_MV)$solutions

##                         post.mean  l-95% CI  u-95% CI  eff.samp pMCMC
## traitlogFood            0.8026629 0.7786648 0.8293304 1000.0000 0.001
## traitlogMass            0.9990370 0.9738648 1.0260262  883.8609 0.001
## traitlogFood:treatment 0.2472409 0.2144592 0.2840184 1000.0000 0.001
## traitlogMass:treatment 0.5117646 0.4780510 0.5509843 1000.0000 0.001
```

Which we can see is almost identical to the estimate from Equation 7

```
c(mean = mean(mod_E7_MV$Sol[, "traitlogFood:treatment"]), SE = sd(mod_E7_MV$Sol[,
    "traitlogFood:treatment"]))

##       mean         SE
## 0.24724086 0.01766363

summary(mod_E7)$coef["treatment", 1:2]

##    Estimate  Std. Error
```

```
## 0.247369313 0.008883709
```

Bivariate models therefore provide an analagous approach to Equation 7, whilst enabling the user to model the presence of measurement error.
